# Academic Primer Series: Eight Key Papers about Education Theory

**DOI:** 10.5811/westjem.2016.11.32315

**Published:** 2017-01-20

**Authors:** Michael Gottlieb, Megan Boysen-Osborn, Teresa M. Chan, Sara M. Krzyzaniak, Nicolas Pineda, Jordan Spector, Jonathan Sherbino

**Affiliations:** * Rush University Medical Center, Department of Emergency Medicine, Chicago, Illinois; † University of California, Irvine, Department of Emergency Medicine, Irvine, California; ‡ McMaster University, Department of Medicine, Division of Emergency Medicine, Hamilton, Ontario, Canada; § University of Illinois, Peoria, Department of Emergency Medicine, Peoria, Illinois; ¶ Universidad San Sebastián, Medicina de Urgencia, Santiago, Chile; || Boston Medical Center, Department of Emergency Medicine, Boston, Massachusetts

## Abstract

**Introduction:**

Many teachers adopt instructional methods based on assumptions of best practices without attention to or knowledge of supporting education theory. Familiarity with a variety of theories informs education that is efficient, strategic, and evidence-based. As part of the Academic Life in Emergency Medicine Faculty Incubator Program, a list of key education theories for junior faculty was developed.

**Methods:**

A list of key papers on theories relevant to medical education was generated using an expert panel, a virtual community of practice synthetic discussion, and a social media call for resources. A three-round, Delphi-informed voting methodology including novice and expert educators produced a rank order of the top papers.

**Results:**

These educators identified 34 unique papers. Eleven papers described the general use of education theory, while 23 papers focused on a specific theory. The top three papers on general education theories and top five papers on specific education theory were selected and summarized. The relevance of each paper for junior faculty and faculty developers is also presented.

**Conclusion:**

This paper presents a reading list of key papers for junior faculty in medical education roles. Three papers about general education theories and five papers about specific educational theories are identified and annotated. These papers may help provide foundational knowledge in education theory to inform junior faculty teaching practice.

## INTRODUCTION

*“But the teacher who has the insight which educational theory affords, inspired is [s]he for excellence in schooling.”*^1^

While educators often hypothesize about the optimal strategies for teaching, the assumed strategies may not always be valid or the most efficient manner for learning. Beliefs may arise from personal experiences or theoretical justifications. In the same manner as in clinical medicine, it is important to use evidence-based and previously tested theories. A firm knowledge of existing education theories provides an educator with the ability to optimize his or her efforts to achieve a desired goal,^2^ thereby minimizing wasted resources on failed or inefficient approaches, improving learning efficiency, and preventing duplication of previously established approaches.

Many academic faculty members are educators, yet few are familiar with key education theories that inform their practice. The *Academic Life in Emergency Medicine* (ALiEM) Faculty Incubator was created in 2016 in an effort to provide advanced education training and mentorship, and to establish a virtual community of practice for early-to-mid-career medical educators. The one-year program consists of a series of modules, each informed by key literature relevant to junior clinician educators. This paper is a narrative review that highlights some of the important literature on the topic of education theory, which was the third module covered in our discovery-based curriculum.

## METHODS

In the third month of the ALiEM Faculty Incubator, the topic of education theory was discussed. We used a method similar to our previous Academic Primer series paper.^3^ We allowed the discussion to unfold and gathered the titles of the papers that were cited, shared, suggested, or discussed within the online discussion forum by both experts and members of this virtual community of practice. This list was then augmented with a general call for suggestions via several social media outlets to optimize our literature list. On Twitter, we tweeted requests to have participants of the #FOAMed and #MedEd online communities provide suggestions for important papers on the topic of educational scholarship within emergency medicine (EM). The Figure shows an exemplar request tweet. All relevant papers discussing education theories were included in the initial analysis.

After the list of key education theory papers was created, a three-round modified Delphi-informed^4^ voting procedure was followed to identify the eight key papers. Voting members included both novices (i.e. early clinician educators) and medical education experts (i.e. experienced clinician educators, all of whom have published greater than 10 peer-reviewed education publications). Novices consisted of Faculty Incubator members who demonstrated particular interest and were the top contributors on the topic of education scholarship, while the experts consisted of the monthly mentors and leaders. The composition of this mixed group was intentional to optimize the identification of articles that represent core content, meet a quality threshold, and are applicable to faculty early in their academic career. Articles were selected using a series of progressive surveys asking the group to rank and then select articles that were both relevant and valuable for early career educators. Once selected, papers were ranked by the percentage of voters who endorsed that this paper “must be included” in the final voting round.

## RESULTS

The initial ALiEM Faculty Incubator discussions identified 20 articles. The one-week social media campaign (May 29, 2016 – June 5, 2016) yielded 18 additional articles. Excluding four duplicates, a total of 34 articles were evaluated.

After initial review of the included articles, the articles were grouped into two broad categories. Eleven articles were categorized as “general theory overview” and 23 articles as “specific theory.” The three-round voting procedure allowed our team to generate a rank-order listing of the papers in order of relevance from the most important to the least important. Five key articles were identified for the “specific theory” category and three for the “general theory overview” category (since there were twice as many articles in the “specific theory” section than the “general theory overview” section). The citations and our ratings of these 34 papers are listed in the Table. After the final eight papers were selected, it was noted that two papers were very similar.^5^,^6^ Therefore, we included only the original paper from 1993 in our discussion and the next highest ranked paper was included as a fifth paper.^7^

## DISCUSSION

The following is a summary of the top eight papers accompanied by commentaries on the relevance of the paper to junior faculty members and considerations for those creating programs for faculty development.

### General Theory Overview

#### 1. Torre DM, Daley BJ, Sebastian JL, Elnicki DM. Overview of current learning theories for medical educators. *Am J Med*. 2006 Oct;119(10):903–7.^8^

##### Summary

This article provides a summary of core learning theories in medical education. The authors briefly discuss behaviorism, cognitive learning theory, humanism, social learning theory, and constructivism. For each of the above approaches, they provide a discussion of the theory itself, followed by potential applications within medical education. In behaviorism, learning is defined by observable behaviors and based upon the relationship between stimulus and response. The teacher plays an active role, while the learner is predominantly a respondent to the imposed environmental stimulus. Cognitive learning theory takes the stance that learning is an internal process. Learners receive, recall, and decode information and form mental representations of it. As they become experts, they create more complex representations (i.e., schemas or semantic networks). To deepen knowledge, defining concepts must be mastered. The teacher must ensure that information is given in an effective and digestible form to learners, so that they may build semantic and conceptual networks. Learners must be more active, as they need to create and reinforce these connections and schema. Two representative examples include the use of concept maps and reflective thinking. In the humanist approach, the goal is to develop a self-directed learner, which is particularly important given the increasing focus on technology in modern education. Social learning theory is based upon modeling and observation of others. Examples include mentorship, role-modeling, and collaborative learning. Finally, constructivism involves the influence of personal experiences to inform (i.e., construct) the interpretation and sense-making of information. Previous experience or knowledge is important when acquiring new information. Learning is goal-oriented. Students learn performing, interacting, and experimenting, while the teacher needs to design, facilitate, and present different tools to learners in learning encounters, for them to build their knowledge.

##### Relevance to Junior Faculty

Educators can improve their teaching efficiency by understanding and using existing education theories. While the core concepts of EM are taught during medical school and residency, the education theories behind successful teaching may not be readily apparent, nor are they typically taught to academic physicians. Without access to these core theories, a medical educator is missing a fundamental element. This can result in educators using inefficient, or even ineffective, teaching techniques. Moreover, given the large number of responsibilities placed on educators and learners, it is important to use proven techniques to maximize the effort and retention by the learner. Education theories can be used to help refine existing education tools, develop new curricula and assessments, and provide the background and rationale for novel teaching innovations. This paper provides an introduction with examples of five common education theories.

##### Considerations for Faculty Developers

This paper may be a useful first resource to provide to junior faculty. As an introductory resource, it compares and contrasts key theories and provides a helpful starting point to allow junior faculty to delve deeper into a theory.

#### 2. Bordage G. Conceptual frameworks to illuminate and magnify. *Med Educ*. 2009 Apr;43(4):312–9.^9^

##### Summary

When encountering a question in education, it is wise to apply a conceptual framework (CF). Bordage compares CF to lighthouses or lenses. A CF illuminates like a lighthouse, and magnifies certain facets of a problem. CFs, therefore, help educators better understand how to approach a problem like a lense. A CF also acknowledges any assumptions made by the investigator in answering the scholarly question. CFs can be well-established theories, models derived from theories, or evidence-based best practices.^9^ Scholars should consider multiple CFs to frame and answer a question, thereby building upon established theory from within or outside one’s field. After selecting the appropriate CF(s), the scholar must rigorously apply the principles of the selected CF.^9^ Thus, the educator is less likely to allow personal bias (i.e., an individual lens) to act as a barrier in identifying novel approaches to solving problems. Bordage presents three vignettes as examples for how to practically apply CFs in practice, thereby demonstrating a step-by-step, educationally sound approach to problems medical educators commonly face.

##### Relevance to Junior Faculty

Scholarship is the currency by which educators advance their career and the field. Junior educators must know how to approach educational problems, design studies in education, and, more broadly, generate scholarship in education. Adequate preparation, which involves conducting a comprehensive literature review and selecting the appropriate CF(s),^9^,^10^ is a key step in designing scholarship. Cook describes CFs as one of six key items to report in educational experiments.^11^ Bordage cites a lack of a CF as a top reason to reject manuscripts in health professions education.^12^ CFs are present in only one-half of published studies in health professions education, but are identified more commonly in journals with higher impact factors.^13^ It is essential that junior educators know how to use conceptual frameworks in designing scholarship.

##### Considerations for Faculty Developers

Bordage asserts that scholarship in health professions education lacks the ubiquitous use of CFs.^9^,^11^ Given that this problem affects many health professions scholars, this should be a priority topic for faculty developers. Thus, faculty developers should have an in-depth understanding of conceptual frameworks. If one considers the issue from a lens of mentorship, knowledge^14^,^15^ of CFs is an essential quality of the successful mentor.

#### 3. Kay D, Kibble J. Learning theories 101: application to everyday teaching and scholarship. *Adv Physiol Educ.* 2016 Mar;40(1):17–25.^16^

##### Summary

This paper uses a problem-solving approach to summarize five major learning theories by applying each to the development of a new curriculum. The paper addresses most of the relevant aspects of the following learning theories: behaviorism, cognitive learning theory, constructivism, social cognitive theory, and social constructivism. In addition to the theories defined above, social cognitive theory expands to include the role of observational learning. Learners will not solely react to stimulus; instead, they will imitate a behavior modeled by others. Social constructivism refers to learning through the internalization and adoption of external experience. Knowledge is acquired by interacting with tools, signs, symbols, and language in the learner’s environment. Optimal learning occurs in a zone of proximal development, where the learner needs a more expert cohort in order to advance. Teachers need to design encounters so that learners face challenges within their zone of proximal development and work together to guide each other. An expert may not be ideal if the expertise is so sophisticated that it is out of the development zone of the learner.

##### Relevance to Junior Faculty

This paper provides a nice summary of major learning theories. It enhances the importance of consistency between the goals, objectives, instructional methods, and assessment strategies chosen for a specific learning activity or course. While there is not one specific theory that applies to every learner, it is valuable to have multiple teaching tools available to effectively reach a spectrum of learners.

##### Considerations for Faculty Developers

This is a great primer for all faculty developers to use when looking to provide an overview paper for new educators. Many of the early career educators in the Faculty Incubator found it difficult to link theory to practice. By providing real-life examples that link these theories to education practice, this paper is able to emphasize the importance of foundational literature.

### Specific Theories

#### 1. Schumacher DJ, Englander R, Carraccio C. Developing the master learner: applying learning theory to the learner, the teacher, and the learning environment. *Acad Med.* 2013 Nov;88(11):1635–45.^17^

##### Summary

As medical educators adopt and implement a competency-based framework in medicine, the authors of this article argue that it is incumbent upon learners to drive their own education and to thoughtfully engage with their teachers and their learning environments to achieve expertise. In essence, competency-based training requires the development of a “master learner.” The authors highlight selected learning theories from the realms of cognitive psychology, experiential learning, and social constructivism, subsequently translating these theories to the clinical learning environment. In a stepwise fashion, this paper introduces the elements of “self-determination theory” (SDT) to describe the processes whereby a master learner derives his or her motivation for learning. Once motivated, elements of “cognitive load theory” (CLT) are introduced, referencing the factors that impact the master learner’s ability to learn in the clinical arena. Through the description of “situated cognition,” we learn how the clinical environment and behaviors of the teachers impact the environment for learning.

##### Relevance to Junior Faculty

This article contains a number of examples to help junior faculty thoughtfully teach their burgeoning master learners. Through an understanding of CLT, junior educators can think through the elements of a task assigned to the learner: providing excessive or too complicated teaching for a learner to process (excessive *germane* load), providing a learning task too large or complicated for a learner to complete (excessive *intrinsic* load), or a task with too many associated elements for a learner to navigate (excessive *extraneous* load).

##### Considerations for Faculty Developers

A motivated learner is one who feels a sense of connection. Faculty developers can cultivate this at a programmatic level by creating a collegial environment where teachers treat learners as though they are on the same team. The authors argue for cohorting trainees on the same treatment team for extended periods, beyond the typical monthly training block, to promote cohesion. Faculty developers should be intimately aware of the state of situated cognition for their learners, exposing learners to teachers whom they admire and seek to emulate. This may include teachers who exemplify the best of evidence-based medical knowledge, superior interpersonal skills, or exceptional teamwork or leadership skills. The authors argue that program chairs should hire faculty who build individual relationships with the learners, and who effectively make tacit thought processes explicit. The work environment should include the physical space and the culture to promote teaching and feedback for learners.

#### 2. Taylor DC, Hamdy H. Adult learning theories: implications for learning and teaching in medical education: AMEE Guide No. 83. *Med Teach.* 2013 Nov;35(11):e1561–72.^18^

##### Summary

This paper provides an overview of a significant number of learning theories in adult education in various contexts. It is included here for its particular attention to Knowles’ theory of andragogy, a widely cited theory in medical education. The components of Knowles’ theory of andragogy include the following: (1) learners need a reason to learn the material; (2) they must have self-concept and be responsible for their own learning; (3) they must have their prior experiences valued; (4) they must have a readiness to learn, (5) they must be oriented to learn; and (6) they must be motivated to learn.

##### Relevance to Junior Faculty

The article provides an overview of theories in adult education, recognizing that adult education theory or andragogy may be foundationally flawed. Of note, Knowles’ theory has been criticized as not being a theory at all, as his work is not informed by experimental or observational data. Moreover, cognitive learning theorists would debate the differences in learning patterns between a child and an adult. The theory also does not consider the importance of context and social factors in acquiring knowledge, skills, and attitudes. However, the principles espoused by Knowles’ theory align with theories and evidence in educational psychology. The arrangement of these principles makes them readily accessible, connecting different concepts into a coherent framework.

##### Considerations for Faculty Developers

Faculty developers should help junior faculty distinguish between true learning theories and seemingly reasonable ad hoc frameworks. The paper ultimately presents a framework that unifies several relevant theories in adult education. The proposed framework may be useful for faculty developers because it attempts to explain the process of learning. Using this framework, faculty developers may be able to design learning environments that promote a better transfer of knowledge, skills, and attitudes to learners in the health professions.

#### 3. Regehr G, Norman GR. Issues in cognitive psychology: implications for professional education. *Acad Med.* 1996 Sep;71(9):988–1001.^19^

##### Summary

Cognitive psychology can be described as the study of how humans think with an intimate linkage to the study of human memory. Although educational and cognitive psychology have historically been viewed as distinct fields, the authors focus on the significant overlap between these philosophies. This paper focuses on five key cognitive psychology concepts that influence our approach to teaching and learning: (1) organization of long-term memory; (2) influences on storage and retrieval from memory; (3) problem solving and transfer, (4) concept formation; and (5) decision making. Specifically, human memory is influenced by the degree to which we can impose meaning on the stimulus, context specificity (i.e., the similarity between the environment in which one learns and retrieves information), processing specificity (i.e., how the storage process will have effect on retrieval), and focused, goal-oriented practice.

##### Relevance to Junior Faculty

These cognitive psychology concepts have implications for the design of curricula and the teaching of learners. Information in isolation is of limited value. Educators should provide opportunities for clinical application of skills and knowledge to strengthen learners’ semantic networks. A learner who has difficulty applying knowledge may not lack understanding, but rather may need to recode the information into a clinically useful form. Additionally, junior faculty should consider the influences on memory when teaching in the clinical environment. Teachers should emphasize clinical and bedside teaching, as well as in situ simulation. In providing variation of the learning and application environments, educators can reduce dependence on context.

##### Considerations for Faculty Developers

This is a valuable primer for faculty developers seeking to provide junior faculty members with an overview of the “basic science” behind teaching and learning (namely, psychology). At times some junior faculty members may be resistant to learning about these new concepts, so it is important for faculty developers to make clear the linkages between these key aspects of psychology and how they relate to a clinical teaching practice.

#### 4. Ericsson KA, Krampe RT, Tesch-Romer C. The role of deliberate practice in the acquisition of expert performance. *Psychological Review.* 1993; 100(3): 363–406.^5^

##### Summary

In this landmark paper, Ericsson et al. provide a thorough description of talent and expertise, arguing that they do not arise from innate skill, but result from a consistent completion of well-planned, closely supervised practice over an extended period of time. The authors coin the term “deliberate practice,” described as activities specially designed to improve the current level of performance, completed frequently over time (with 10 years described as an optimal duration). The authors examine a variety of contexts in which expertise has been described, including chess, art, athletics, and typing. They provide a multitude of references to support the idea that individuals are not born experts; rather, they often display a certain affinity for an activity at a young age, and begin to perform it earlier and more often than those with less “talent”. The authors reference a number of studies that dispel the notion that hereditary factors confer an increased likelihood of expertise in any particular domain. Alongside the comprehensive examination of expertise as a result of practice, this paper includes two primary studies of musicians, comparing the study habits of “expert performers”. They argue that the difference between an expert and a good musician is likely the result of more frequent practice (and less non-music focused leisure) over the many years of musical training.

For further reading on this theory, we highly recommend this article to both junior faculty members and faculty developers: Ericsson KA. acquisition and maintenance of medical expertise. *Acad Med.* 2015 Nov;90(11):1471–86.^6^

##### Relevance to Junior Faculty

The fundamental tenet of this paper is that expertise is acquired rather than inherited. It follows that any teacher can foster expertise in their learners via deliberate practice. It is important to note that not all practice is deliberate practice. For example, sending medical students home with a pig’s foot and asking them to practice suturing 100 times by themselves does not satisfy the criteria of deliberate practice. It requires that the medical student “be given explicit instructions about the best method and be supervised by a teacher to allow individualized diagnosis of errors, informative feedback, and remedial part training.”^5^ The learner should be asked to repeatedly complete the task, with the instructor consistently available to correct and refine. Simulation facilitates the effective deployment of deliberate practice because it allows for frequent repetition not necessarily experienced in the unpredictable authentic clinical environment.

##### Considerations for Faculty Developers

Faculty developers must consider the great deal of time and effort required by deliberate practice. The individualized learning exercises must be unique to the learner and closely supervised. For the educator teaching a large number of learners or simultaneously tending to multiple levels of learners, deliberate practice would be challenging because of the individualized attention required. Finally, deliberate practice requires full attention and effort, which can only be sustained for a finite period of time for most learners and may require recovery time between sessions.

#### 5. Young JQ, Van Merrienboer J, Durning S, Ten Cate O. Cognitive load theory: implications for medical education: AMEE Guide No. 86. *Med Teach.* 2014 May;36(5):371–84.^7^

##### Summary

This paper, from the classic AMEE Guide series, summarizes cognitive load theory. This theory is informed by models of human memory that suggest that sensory, working, and long-term memory are interlinked. Working memory has a very finite capacity, which is the rate-limiting step for learning. This paper defines key terms such as intrinsic load, extraneous load, and germane load and applies them to the learner.

##### Relevance to Junior Faculty

For junior faculty members, this paper is a key primer to understanding the science and theory behind learning. Understanding the different components of human memory and capacity is invaluable when teaching the learner, emphasizing high-yield learning and avoiding extraneous cognitive load.

##### Considerations for Faculty Developers

This paper serves as a good overview of a very rich area of cognitive science. The paper is admittedly quite dense, so it would be prudent to guide new educators through this paper with clinical- or classroom-specific examples to bring these concepts to life.

## LIMITATIONS

Similar to our previous ALiEM Academic Primer Series papers, the main limitation is that we did not use a systematic or comprehensive search strategy. However, we did attempt to triangulate recommendations for key literature from multiple sources (e.g. Faculty Incubator discussions, Twitter, etc.). Additionally, while we did attempt to provide a broad range of inputs, there is potential for bias, as most of the submissions were from a limited number of junior faculty and experts. We did augment this by using multiple social media calls, which resulted in a large number of additional suggestions.

## CONCLUSION

This paper presents a reading list of key papers for junior faculty in medical education roles. Three papers about general education theories and five papers about specific educational theories are identified and annotated. These papers may help provide foundational knowledge in education theory to inform junior faculty teaching practice.

## Figures and Tables

**Figure:**
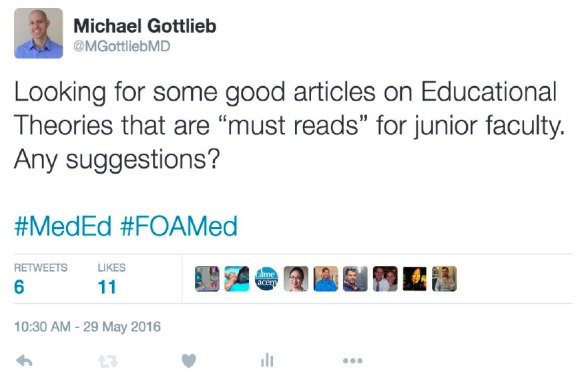
Example of an exemplar Tweet used in the process of helping to generate a list of key papers on theories relevant to medical education.

**Table:** The complete list of educational scholarship literature collected by the authorship team.

Citation	Round 1 initial mean scores (SD) max score 7	Round 2 % of raters that endorsed this paper	Round 3 % of raters that endorsed paper in last round	Top 3 overview papers	Top 5 papers describing a key education theory
Torre DM, Daley BJ, Sebastian JL, et al. Overview of current learning theories for medical educators. *Am J Med*. 2006 Oct;119(10):903–7.^8^	6.4 (0.8)	85.7%	100% endorsed as a good overview	1	
Bordage G. Conceptual frameworks to illuminate and magnify. *Med Educ*. 2009 Apr;43(4):312–9.^9^	6.1 (1.6)	85.7%	85.7% endorsed as a good overview	2	
Schumacher DJ, Englander R, Carraccio C. Developing the master learner: applying learning theory to the learner, the teacher, and the learning environment. *Acad Med*. 2013 Nov;88(11):1635–45.^17^	6.1 (1.1)	85.7%	85.7% endorsed as a key paper with important educational theories to know		1
Taylor DC, Hamdy H. Adult learning theories: implications for learning and teaching in medical education: AMEE Guide No. 83. *Med Teach*. 2013 Nov;35(11):e1561–72.^18^	5.9 (1.5)	71.4%	85.7% endorsed as a key paper with important educational theories to know		2
Kay D, Kibble J. Learning theories 101: application to everyday teaching and scholarship. *Adv Physiol duc*. 2016 Mar;40(1):17–25.^16^	5.7 (1.5)	71.4%	57.1% endorsed as a good overview	3	
Young JQ, Van Merrienboer J, Durning S, et al. Cognitive Load Theory: implications for medical education: AMEE Guide No. 86. *Med Teach*. 2014 May;36(5):371–84.^7^	5.7 (1.4)	42.9%	28.6% endorsed as a key paper with important educational theories to know		5
Ericsson KA. Acquisition and Maintenance of Medical Expertise. *Acad Med*. 2015 Nov;90(11):1471–86.^6^	5.6 (1.6)	57.1%	57.1% endorsed as a key paper with important educational theories to know		4^*^
Ericsson KA, Krampe RT, Tesch-Romer C. The Role of Deliberate Practice in the Acquisition of Expert Performance. *Psychological Review*. 1993; 100(3): 363–406.^5^	5.6 (1.1)	42.9%	57.1% endorsed as a key paper with important educational theories to know		4
Regehr G, Norman GR. Issues in cognitive psychology: implications for professional education. *Acad Med*. 1996 Sep;71(9):988–1001.^19^	5.4 (1.1)	71.4%	7.14% endorsed as a key paper with important educational theories to know		3
Eva KW, Regehr G. “I’ll never play professional football” and other fallacies of self-assessment. *J Contin Educ Health Prof*. 2008 Winter;28(1):14–9.^20^	5.3 (1.4)	57.1%			
Kaufman DM. Applying educational theory in practice. *BMJ*. 2003 Jan 25; 326(7382): 213–216.^21^	5.4 (1.4)	28.6%			
Miller GE. The assessment of clinical skills/ competence/ performance. *Acad Med*. 1990 Sep;65(9 Suppl):S63–7.^22^	5.4 (1.3)	28.6%			
Li LC, Grimshaw JM, Nielsen C, et al. Evolution of Wenger’s concept of community of practice. *Implement Sci*. 2009 Mar 1;4:11.^23^	5.3 (1.4)	14.3%			
Mann KV. Theoretical perspectives in medical education: past experience and future possibilities. *Med Educ*. 2011 Jan;45(1):60–8.^24^	5.3 (1.7)	57.1%			
Schuwirth LWT, Van der Vleuten CPM. General overview of the theories used in assessment: AMEE Guide No 57. *Med Teach*. 2011;33(10):783–97.^25^	5.3 (1.7)	57.1%			
Flynn L, Jalali A, Moreau KA. Learning theory and its application to the use of social media in medical education. *Postgrad Med J*. 2015 Oct;91(1080):556–60.^26^	5.1 (1.3)	42.9%			
Wolff M, Wagner MJ, Poznanski S, et al. Not another boring lecture: engaging learners with active learning techniques. *J Emerg Med*. 2015 Jan;48(1):85–93.^27^	5.0 (1.5)	28.6%			
Sandars J, Cleary TJ. Self-regulation theory: applications to medical education: AMEE Guide No. 58. *Med Teach*. 2011;33(11):875–86.^28^	5.0 (1.4)	28.6%			
Azer SA, Guerrero AP, Walsh A. Enhancing learning approaches: practical tips for students and teachers. *Med Teach*. 2013 Jun;35(6):433–43.^29^	4.7 (1.0)	42.9%			
Mughal F, Zafar A. Experiential Learning from a Constructivist Perspective-Reconceptualizing the Kolbian Cycle. *International Journal of Learning and Development.* 2011;1(2):27–37.^30^	4.6 (1.0)	42.9%			
Kuper A, Whitehead C. The practicality of theory. *Acad Med*. 2013 Nov;88(11):1594–5.^31^	4.6 (1.6)	14.3%			
Pangaro L. A new vocabulary and other innovations for improving descriptive in-training evaluations. *Acad Med*. 1999 Nov;74(11):1203–7.^32^	4.6 (1.3)	0%			
Yardley S, Teunissen PW, Dornan T. Experiential learning: AMEE Guide No. 63. *Med Teach*. 2012;34(2):e102–15.^33^	4.6 (1.1)	42.9%			
Watling CJ, Lingard L. Grounded theory in medical education research: AMEE Guide No. 70. *Med Teach*. 2012;34(10):850–61.^34^	4.4 (2.0)	14.3%			
Norman GR. The adult learner: a mythical species. *Acad Med*. 1999 Aug;74(8):886–9.^35^	4.3 (2.0)	42.9%			
Wong G. Literature reviews in the health professions: It’s all about the theory. *Med Educ*. 2016 Apr;50(4):380–2.^36^	4.0 (1.0)	14.3%			
Norman GR. Problem solving skills, solving problems and problem-based learning. *Med Educ*. 1988 Jul;22(4):279–86.^37^	4.0 (1.2)	0%			
Santen SA, Deiorio NM, Gruppen LD. Medical education research in the context of translational science. *Acad Emerg Med*. 2012 Dec;19(12):1323–7.^38^	3.9 (1.5)	0%			
McGaghie WC. Medical education research as translational science. *Sci Transl Med*. 2010 Feb 17;2(19):19cm8.^39^	3.6 (2.1)	0%			
Norman G. Data dredging, salami-slicing, and other successful strategies to ensure rejection-twelve tips on how to not get your paper published. *Adv Health Sci Educ Theory Pract*. 2014 Mar;19(1):1–5.^40^	3.4 (1.5)	14.3%			
Zerzan JT, Hess R, Schur E, et al. Making the most of mentors: a guide for mentees. *Acad Med*. 2009 Jan;84(1):140–4.^41^	3.1 (1.8)	28.6%			
Grow G. Teaching learners to be self-directed. *Journal of Adult Education Quarterly Spring*. 1991; 41:125–149.^42^	3.1 (2.1)	14.3%			
Azer SA. The top-cited articles in medical education: a bibliometric analysis. *Acad Med*. 2015 Aug;90(8):1147–61.^43^	2.7 (2.2)	42.9%			
Sherbino J, Kulasegaram K, Worster A, et al. The reliability of encounter cards to assess the CanMEDS roles. *Adv Health Sci Educ Theory Pract*. 2013 Dec;18(5):987–96.^44^	2.6 (1.3)	0%			
